# Histopathological Study of Regenerative Endodontic Therapy on an Immature Mandibular Second Premolar With Pulp Necrosis: A Case Report

**DOI:** 10.7759/cureus.95647

**Published:** 2025-10-29

**Authors:** Hidefumi Sako, Kazuhiro Omori, Yuki Shinoda-Ito, Kiyofumi Takabatake, Hitoshi Nagatsuka, Shogo Takashiba

**Affiliations:** 1 Department of Periodontics and Endodontics, Division of Dentistry, Okayama University Hospital, Okayama, JPN; 2 Private Practice, Marumo Dental Clinic, Gifu, JPN; 3 Department of Pathophysiology - Periodontal Science, Faculty of Medicine, Dentistry and Pharmaceutical Sciences, Okayama University, Okayama, JPN; 4 Department of Oral Pathology, Faculty of Medicine, Dentistry and Pharmaceutical Sciences, Okayama University, Okayama, JPN

**Keywords:** calcium hydroxide, immature permanent teeth, pulp necrosis, regenerative endodontic therapy, revascularization

## Abstract

Regenerative endodontic therapy (revascularization) for immature permanent teeth with pulp necrosis and/or apical periodontitis is an effective treatment to promote root maturation. Previous histological studies have reported the formation of cementoid or osteoid tissue and periodontal ligament-like tissue within the root canals. This case report presents the histopathological findings of a human immature permanent tooth with pulp necrosis following revascularization.

A 11-year-old male patient presented with tenderness on biting and the formation of a sinus tract in the mandibular right second premolar (tooth #29), diagnosed as pulp necrosis with symptomatic apical periodontitis. Revascularization was performed using calcium hydroxide as an intracanal medicament, with reference to the American Association of Endodontists (AAE) 2018 Position Paper on Regenerative Endodontics. At the 12-month follow-up, radiographs showed thickening of the canal walls, apical narrowing, root elongation, and recovery of pulp sensibility. The tooth was later extracted for orthodontic reasons at 42 months and processed for histological examination.

Histological evaluation revealed cementum-like hard tissue continuous with the existing dentin in the apical region, suggesting apical closure. In contrast, the coronal portion showed less mature cementum-like tissue accompanied by loose connective tissue and neovascularization. These findings indicate that revascularization with calcium hydroxide can induce the formation of cementum-like and dentin-like tissues with vascular regeneration in immature permanent teeth with pulp necrosis.

## Introduction

Management of immature permanent teeth with necrotic pulp presents a considerable clinical challenge. Specifically, young teeth lacking vitality and characterized by open apices exhibit thin and fragile canal walls, complicating both debridement and the achievement of an adequate apical seal with conventional endodontic techniques. Traditionally, apexification with calcium hydroxide or mineral trioxide aggregate (MTA) has been the primary treatment option for such cases [1]. However, apexification does not address the problem of halted root development or the resulting thin, fragile root walls [2].

Regenerative endodontic therapy (RET), called revascularization, was introduced by Iwaya et al. [3] and Banchs and Trope [4] for immature non-vital teeth. Their cases showed that a human necrotic immature permanent tooth could experience increased root canal wall thickening and continued root development after revascularization. Radiographically, revascularized immature permanent teeth exhibited apical closure, root elongation, and canal wall thickening, which are considered indirect indicators of healing after regenerative endodontic procedures. However, histologic studies of revascularized teeth in animal models have shown that the newly formed tissue within the pulp space mainly consists of cementum-like, osteoid-like, and/or periodontal ligament-like tissues [5-8], regardless of the presence or absence of apical lesions and irrespective of the use of specific stem cells, scaffolds, or growth factors. Most clinical investigations on immature teeth with pulp necrosis and apical periodontitis treated by revascularization have reported that the tissues generated within the canal space were similar to those observed in animal models [9-14]. Moreover, the standard revascularization procedure involves promoting bleeding from the root canal during the second appointment, following cleaning and calcium hydroxide application, then placing sufficiently thick MTA over the unstable blood clots to seal the canal [3]. However, this technique is highly challenging.

In this case report, we demonstrate an instance where an immature mandibular right second premolar (tooth #29) with pulp necrosis due to fracture of the dens evaginatus was successfully healed after revascularization using calcium hydroxide preparation without MTA. This report also includes the histological observation of a human tooth following a revascularization procedure.

This article was previously presented as a poster at the 159th Japanese Society of Conservative Dentistry Annual Meeting on November 12, 2023.

## Case presentation

A 11-year-old male patient with no relevant medical history experienced tenderness on biting and swelling of the buccal gingiva at tooth #29 in May 2019. He visited the family dental clinic and underwent radiographic examination. After a panoramic radiograph, it was noted that there was a significant radiolucent area around the root apex of tooth #29, and the family dentist explained that normal endodontic treatment would be difficult. As a result, the patient was referred from the family dentist to the Department of Periodontics and Endodontics, Division of Dentistry, Okayama University Hospital, for endodontic treatment of tooth #29 in July 2019.

First treatment visit

**Figure 1:**
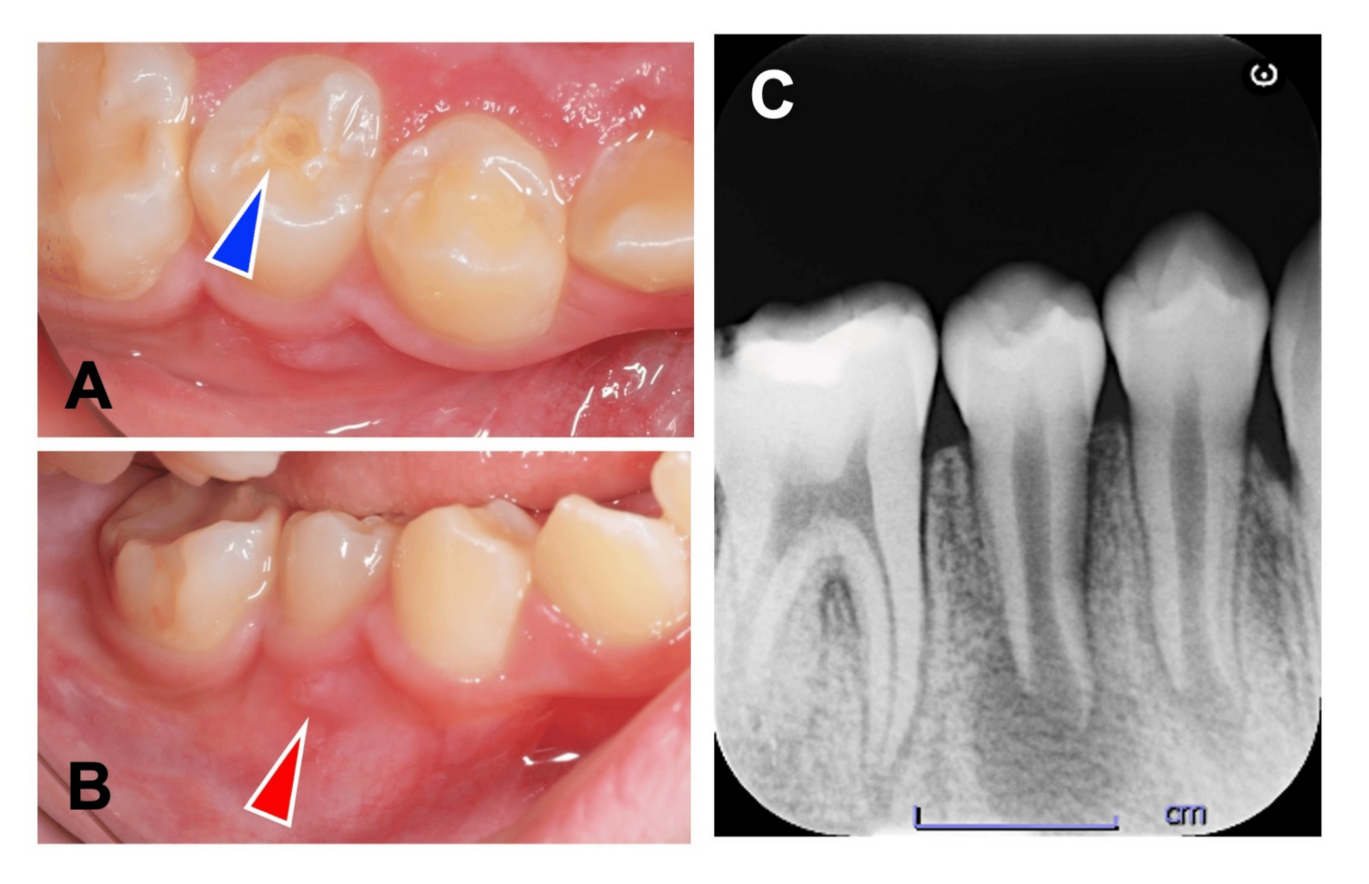
Preoperative clinical and radiographic features of tooth #29. (A) A photograph of tooth #29 with a fractured dens evaginatus. The blue arrowhead indicates the site of fractured dens evaginatus. (B) A photograph of a sinus tract formed on the buccal side of tooth #29. The red arrowhead indicates the site of sinus tract formation. (C) Preoperative radiograph of tooth #29 demonstrating incomplete root formation accompanied by periapical radiolucency.

A fractured dens evaginatus was observed in tooth #29 (Figure 1A), accompanied by percussion pain and no response to thermal testing (Pulper Dental Coolant, GC Corporation, Tokyo, Japan) or electric pulp testing (Pulptester, Yoshida, Tokyo, Japan). A trace of a sinus tract was present in the buccal gingiva (Figure 1B). Radiographic examination showed an open apex in tooth #29 with notable periradicular radiolucency (Figure 1C). The diameter of the apical foramen was 2.5 mm. The root length, measured from the cementoenamel junction (CEJ) to the root apex, was 14.7 mm. The tooth was diagnosed as having pulp necrosis associated with symptomatic apical periodontitis and was classified as Cvek's stage 3. Since tooth #29 was immature, we determined that revascularization, in reference to the American Association of Endodontists (AAE) 2018 Position Statement [15], was more appropriate than apexification. Explanations regarding the indications, techniques, and relative advantages and disadvantages of apexification and revascularization were provided to the patient and his mother. Revascularization was selected, and informed consent was obtained prior to initiating therapy.

Although the pulp was non-vital, the patient experienced pain during the treatment; therefore, the following procedures were performed under local anesthesia. After infiltrating local anesthesia with 2% lidocaine containing 1/80,000 epinephrine (ORA Injection Dental Cartridge, GC Showa Yakuhin, Tokyo, Japan) and isolating with a rubber dam, the access cavity was opened. Upon puncturing the pulp cavity, bleeding and drainage occurred. Most of the pulp tissue was necrotic, and the necrotic tissue was removed with a K-file (#90, stainless steel; MANI, Tochigi, Japan). The root canal was cleaned with Neo Cleaner (10% NaOCl; Neo Dental Chemical Products, Tokyo, Japan) and Smear Clean (3% ethylenediaminetetraacetic acid (EDTA); Nippon Shika Yakuhin, Yamaguchi, Japan) using active irrigation with a 15 mL disposable syringe and a closed-ended, side-vented needle. Although the concentrations differed from those recommended by the AAE (1.5-3% NaOCl and 17% EDTA), these reagents were selected because they are commonly used and commercially available in Japan for root canal disinfection. The working length was measured from the crown to the radiographic root apex on dental radiographs and set at 18 mm, 1 mm short of the radiographic apex, to determine the appropriate depth for needle insertion and to prevent apical extrusion of the irrigant. Calcipex Plane II (CP II; calcium hydroxide water-based paste, Nippon Shika Yakuhin) was applied to the working length to sterilize the root canal. Glass ionomer cement (GIC; Fuji II LC, GC Corporation) was then used for a tight temporary seal.

Second treatment visit

**Figure 2:**
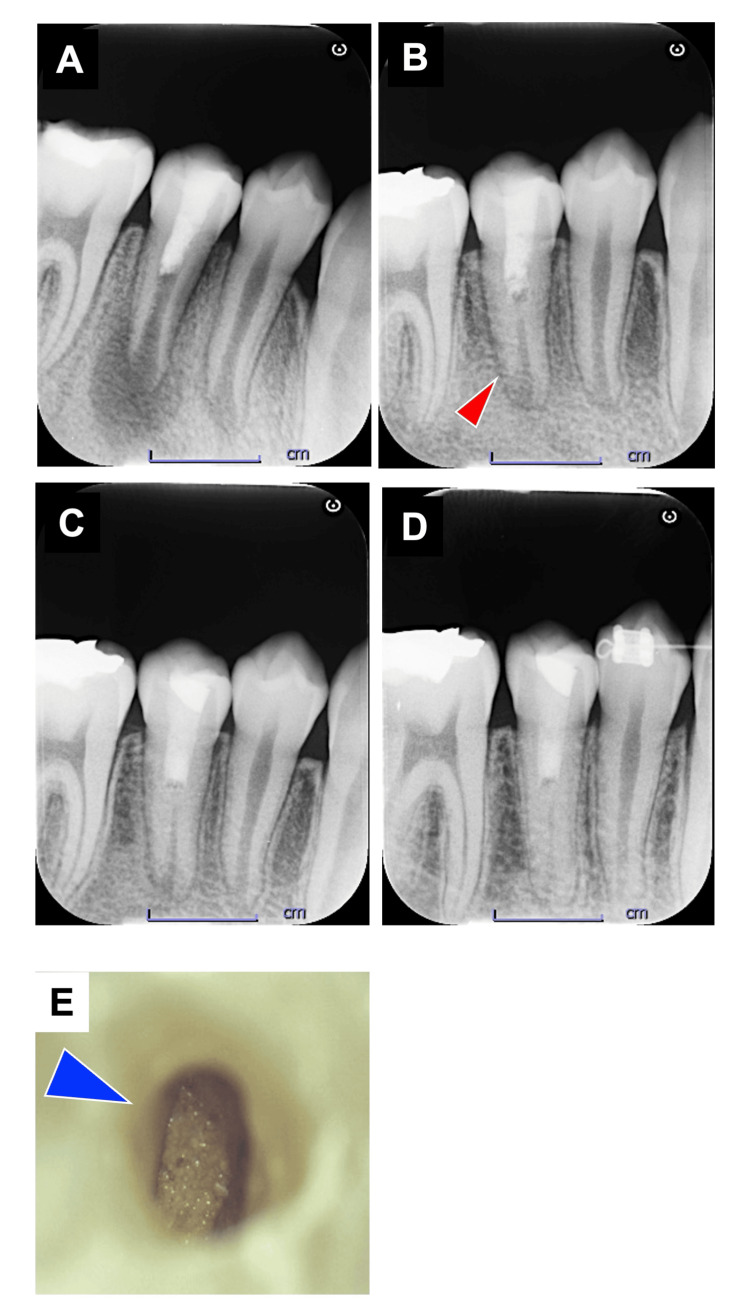
Post-revascularization radiographic progression and hard tissue interface formation in tooth #29. (A) A radiograph of tooth #29 immediately after revascularization. (B) Three months postoperatively, the radiograph demonstrates marked canal wall thickening and apical constriction, with no evidence of periapical radiolucency. (C) At seven months after treatment, following definitive restoration with composite resin, the radiograph reveals further reinforcement of the canal walls and continued narrowing at the apex, without periapical radiolucency. (D) The 12-month follow-up radiograph of tooth #29 shows pronounced canal wall apposition and apical foramen closure, again without signs of periapical radiolucency. (E) A photograph of the interface after removing the calcium hydroxide paste, seven months post-revascularization. The formation of a hard tissue-like interface was confirmed.

After one week following the first visit, tooth #29 showed no percussion pain, the gingival swelling had reduced, and the sinus opening was absent. Local infiltration anesthesia with 2% lidocaine without a vasoconstrictor (Scandonest cartridge, Nippon Shika Yakuhin) was administered. Under rubber dam isolation, CP II paste was removed with sterile saline irrigation. The canal was then irrigated with Neo Cleaner and saline and dried with sterile paper points (Zipperer®, VDW GmbH, Munich, Germany). The K-file (#15) was inserted 2 mm beyond the root apex to induce bleeding into the canal. Root canal bleeding stopped approximately 4.5 mm below the CEJ (measured with a pocket probe). CP II was applied over the dark red blood clots formed up to the CEJ. The access cavity was sealed with GIC. The radiographic examination showed that the root apex was open, and there was a distinct periapical radiolucency around it (Figure 2A).

Third treatment visit

At the three-month recall (October 2019), clinical examination of tooth #29 showed no symptoms. The tooth was not sensitive to percussion pain, and the gingival swelling had resolved. No response was detected to thermal or electric pulp testing. Radiographic evaluation indicated limited root elongation in the absence of canal wall thickening.

Fourth treatment visit

At the seven-month recall (February 2020), there was no percussion pain, and the tooth showed a positive response to the electric pulp tester, which had shown no response at the first visit. Radiographic examination revealed the formation of radiopaque tissues approximately 2 mm beneath the placement site of CP II, extending along the canal wall toward the apical region. Additionally, the disappearance of the apical radiolucency, closure of the apical foramen, and lengthening of the root were confirmed (Figure 2B). The diameter of the apical foramen was 2 mm, and the root length was 16.4 mm. When CP II was removed from the root canal, calcified tissue formation was observed at the interface (Figure 2E). A final restoration was made from the surface of the hard tissue formation to the crown using Clearfill® DC Core Automix® ONE and Majesty® ES Flow (Kuraray Noritake Dental, Niigata, Japan).

Follow-up visits

At the 12-month recall (July 2020), the patient was progressing well after the composite restoration. There was a positive response to an electric pulp tester. Radiographic examination confirmed a tendency for the root canal to narrow, with closure of the apical foramen and root elongation (Figure 2C). The root canal diameter measured 1.2 mm, and the root length was 16.7 mm.

At the 36-month recall (July 2022), no clinical symptoms were observed, confirming the successful outcome of the treatment with a positive response to an electric pulp tester. Radiographic examination revealed that the root apex diameter had decreased to 0.6 mm, and the root length had increased to 17.3 mm (Figure 2D). The root apex morphology was similar to that of tooth #28, and the formation of the periodontal ligament space consistent with the root apex morphology was also observed. An intervening radiolucent area between the restoration and the formed hard tissue was confirmed.

Histologic procedure

At the 42-month recall (February 2023), tooth #29 with a positive response to the electric pulp tester was scheduled for extraction due to orthodontic reasons. Immediately after extraction, the tooth was fixed in 4% paraformaldehyde for 48 hours, decalcified in 10% EDTA (pH 7.5) at 4°C for four weeks, dehydrated in ethanol, and embedded in paraffin. Serial longitudinal sections of 5 μm were prepared, stained with hematoxylin and eosin (HE), and examined under a light microscope (BZ-X800, KEYENCE, Osaka, Japan).

Histologic observation

**Figure 3:**
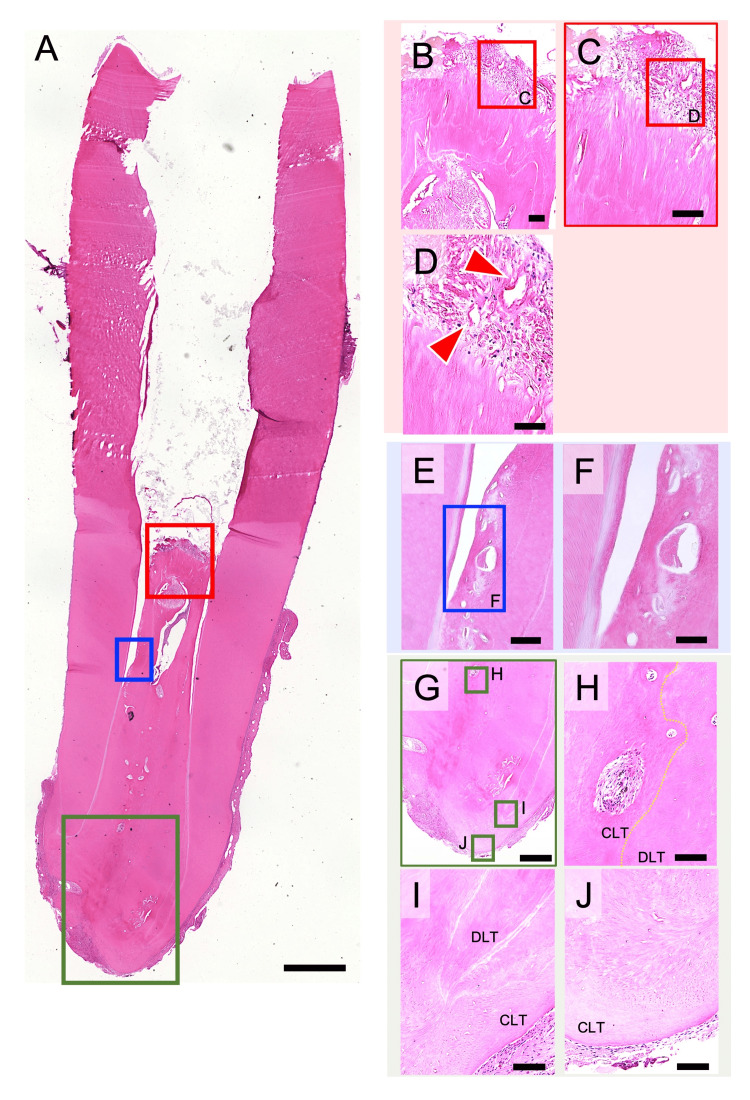
Histological evaluation of regenerated dentin and cementum-like tissues after revascularization of tooth #29. (A) A section taken through the central portion of the root canal of tooth #29, showing newly generated connective tissue and mineralized deposits along the canal walls (hematoxylin and eosin, original magnification ×2). (B-D) A detailed image of the upper red rectangle in A shows the regenerated soft tissue as uninflamed fibrous connective tissue characterized by spindle-shaped fibroblasts and collagen fibers interspersed with blood vessels (red arrowhead). This tissue fills the canal space up to the coronal calcium hydroxide (original magnification, B: ×40; C: ×100, D: ×200). (E, F) A detailed image of the middle blue rectangle in A reveals regenerated hard tissue along the canal walls, consisting of both cellular and acellular cementum-like tissue. Numerous cementocyte-like cells are observed within the cellular component (original magnification: E, ×100; F, ×200). (G-J) A detailed image of the lower green rectangle in A shows neointimal cementum-like hard tissue deposited adjacent to the pre-existing dentin, with a tendency toward apical closure (original magnification: G, ×40; H-J, ×100). CLT, cementum-like tissue; DLT, dentin-like tissue. Scale bars: A, 2 mm; B and C, 100 μm; D, 50 μm; E, 100 μm; F, 50 μm; G, 500 μm; H-J, 100 μm.

Histologically, there was a space extending up to the upper half of the root canal. This may be due to the formation of a calculus-like hard tissue (Figure 2E) at the interface with the composite resin filled in the root canal, which was detached during specimen preparation (Figure 3A). The formation of new cementum-like hard tissue was observed in the lower half of the root canal. Compared to the apex side, the new cementum-like hard tissue on the coronal side was immature, with fibroblast-like and vascular endothelial-like cells, as well as angiogenesis, observed in the superficial and subapical soft tissues (Figures 3B-3D). On the apex side, cementum-like hard tissue formation was present in addition to the existing dentin, and the root apex tended to close (Figures 3G-3J). There was also a lumen containing cellular components; however, no cellular components were observed at the interface between the new cementum-like tissue and the existing dentin (Figures 3E-3F). The increased root length and thickness resulted from apical deposition of the newly formed cementum-like tissue.

## Discussion

The present case demonstrated that RET, revascularization, using calcium hydroxide induced the formation of pulp-like, dentin-like, and cementum-like tissues in a necrotic immature premolar with an apical abscess. Calcium hydroxide is well recognized for its antimicrobial efficacy in infected root canals [16] and is frequently employed as an intracanal medicament during revascularization procedures in immature permanent teeth with necrotic pulp and apical pathology [17]. It was selected in the present case to achieve effective disinfection while minimizing the risk of tooth discoloration and cytotoxic effects on apical stem cells, which have been reported with the use of triple antibiotic paste. Moreover, a recent meta-analysis reported that calcium hydroxide tends to promote greater apical closure, whereas triple antibiotic paste is more frequently associated with increased dentin wall thickness, reflecting their distinct biological effects [18]. MTA was not used as an intracanal medicament but was reserved for coronal sealing after the induction of bleeding. In this case, no vital tissue remained in the root canal before treatment, and healing was achieved using only a blood clot as a scaffold without the addition of exogenous cells such as platelet-rich plasma (PRP).

Histological analysis revealed that the healing response did not represent full regeneration of the pulp-dentin complex, but was instead dominated by the deposition of dentin- or cementum-like tissue (Figure 3). This finding is consistent with previous studies reporting that protocols based on induced apical bleeding are often associated with pulp canal obliteration (PCO) or canal calcification [19]. Although the clinical significance of such calcification remains controversial, evidence indicates that the incidence of PCO is lower when alternative scaffolds such as PRP gels are used [20]. These findings underscore the need for further studies to clarify the biological role and clinical implications of calcification and to optimize scaffold selection in regenerative endodontics.

Another important observation was the presence of granular structures of uncertain origin (Figure 2E). Because modern regenerative endodontic procedures and vital pulp therapies are typically performed using single-visit protocols with materials such as MTA, opportunities to examine re-entry specimens are limited, resulting in a scarcity of published data. The granular deposits identified in this case differed from calcium carbonate precipitates previously associated with calcium hydroxide use and may instead represent cementum-related tissue. Further pathological and surgical investigations are warranted to determine their precise nature.

In addition, a gap was observed between the pre-existing dentin and the newly formed mineralized tissues (Figures 3E-3F). It is plausible that this space was not entirely empty but contained fibroblast-like cells or other connective tissue elements. Similar findings have been reported in traumatized teeth, where PCO occurs as part of transient apical breakdown [21]; however, histological comparisons between these conditions are lacking. A further noteworthy point in the present case is that the healing pattern resembled that described in a recent report [22], in which endodontic surgery was performed for recurrent apical periodontitis after revascularization, and the resected regenerated root apex was examined by scanning electron microscopy (SEM) [21]. A previous case report has also performed immunohistochemical analysis of revascularized root canals and demonstrated high periostin protein expression, suggesting an active reparative process rather than true regeneration [23]. Therefore, future studies incorporating immunohistochemical analysis and high-resolution ultrastructural evaluation using SEM are expected to clarify the healing mechanisms of revascularization and thereby contribute to improving its success rate.

## Conclusions

Revascularization with a calcium hydroxide preparation was performed on an immature mandibular premolar with pulp necrosis caused by a fracture of dens evaginatus, resulting in good extension of the root canal wall and root in this case. Histologic analysis of the tooth specimens confirmed the formation of granular hard tissue and pulp-like tissue, including blood vessels, directly beneath the calcium hydroxide preparation, as well as the formation of new cementum-like tissue and the restoration of the root apex morphology. This case report may be very useful in understanding the healing mechanism of endodontic revascularization.
